# Cyproheptadine, an antihistaminic drug, inhibits proliferation of hepatocellular carcinoma cells by blocking cell cycle progression through the activation of P38 MAP kinase

**DOI:** 10.1186/s12885-015-1137-9

**Published:** 2015-03-17

**Authors:** Yu-Min Feng, Chin-Wen Feng, Syue-Yi Chen, Hsiao-Yen Hsieh, Yu-Hsin Chen, Cheng-Da Hsu

**Affiliations:** 1 Department of Internal Medicine, Ditmanson Medical Foundation Chia-Yi Christian Hospital, Chia-Yi, Taiwan; 2 Department of Medical Research, Ditmanson Medical Foundation Chia-Yi Christian Hospital, Chia-Yi, Taiwan; 3 Department of Biological Science, National Sun Yat-sen University, Kaohsiung, Taiwan

**Keywords:** Hepatocellular carcinoma, Cyproheptadine, Cell cycle arrest, Apoptosis, p38 MAP kinase

## Abstract

**Background:**

Hepatocellular carcinoma (HCC) is a major cause of cancer deaths worldwide. However, current chemotherapeutic drugs for HCC are either poorly effective or expensive, and treatment with these drugs has not led to satisfactory outcomes. In a 2012 case report, we described our breakthrough finding in two advanced HCC patients, of whom one achieved complete remission of liver tumors and the other a normalized α-fetoprotein level, along with complete remission of their lung metastases, after the concomitant use of thalidomide and cyproheptadine. We assumed the key factor in our effective therapy to be cyproheptadine. In this study, we investigated the antiproliferative effects and molecular mechanisms of cyproheptadine.

**Methods:**

The effect of cyproheptadine on cell proliferation was examined in human HCC cell lines HepG2 and Huh-7. Cell viability was assayed with Cell Counting Kit-8; cell cycle distribution was analyzed by flow cytometry. Mechanisms underlying cyproheptadine-induced cell cycle arrest were probed by western blot analysis.

**Results:**

Cyproheptadine had a potent inhibitory effect on the proliferation of HepG2 and Huh-7 cells but minimal toxicity in normal hepatocytes. Cyproheptadine induced cell cycle arrest in HepG2 cells in the G1 phase and in Huh-7 cells at the G1/S transition. The cyproheptadine-induced G1 arrest in HepG2 cells was associated with an increased expression of HBP1 and p16, whereas the G1/S arrest in Huh-7 cells was associated with an increase in p21 and p27 expression and a dramatic decrease in the phosphorylation of the retinoblastoma protein. Additionally, cyproheptadine elevated the percentage of Huh-7 cells in the sub-G1 population, increased annexin V staining for cell death, and raised the levels of PARP and its cleaved form, indicating induction of apoptosis. Finally, cyproheptadine-mediated cell cycle arrest was dependent upon the activation of p38 MAP kinase in HepG2 cells and the activation of both p38 MAP kinase and CHK2 in Huh-7 cells.

**Conclusions:**

Our results demonstrate that a non-classical p38 MAP kinase function, regulation of cell cycle checkpoints, is one of the underlying mechanisms promoted by cyproheptadine to suppress the proliferation of HCC cells. These results provide evidence for the drug’s potential as a treatment option for liver cancer.

**Electronic supplementary material:**

The online version of this article (doi:10.1186/s12885-015-1137-9) contains supplementary material, which is available to authorized users.

## Background

Hepatocellular carcinoma (HCC) is the predominant primary liver cancer, with over half a million new cases diagnosed annually [1], and is the fifth most frequently diagnosed cancer worldwide [2]. The very poor prognosis of HCC makes it the second leading cause of cancer-related death, corresponding to an estimated 695,900 deaths annually [2]. In most countries, HCC accounts for 70%–85% of primary liver cancer cases [3]. In Taiwan, HCC has an incidence of approximately 10,000 new cases per year and has been the leading cause of cancer death for the past two decades [4]. HCC is frequently asymptomatic in its early stages; thus, almost 85% of patients diagnosed with HCC are in intermediate or advanced stages, for which limited treatment options are available [5,6]. Despite extensive application of targeted therapy, current treatment for advanced HCC is still not satisfactory [7]. Therefore, there have been continued interest and active research in developing effective targeted agents for HCC.

Molecular studies in recent years have highlighted various potential therapeutic targets in HCC, including VEGF and FGF, EGFR, HGFR/c-Met, IGFR, survivin, Wnt signaling, Src signaling, the Ras/Raf/p38 MAP kinase (MAPK) pathway, and the PI3K/AKT/mTOR pathway [6,8,9]. As a result, a wide range of novel targeted agents for advanced HCC have been developed or are under development. Although the VEGF-targeted agent sorafenib (Nexavar, Bayer Pharmaceuticals) has been shown to have a clinically meaningful overall survival benefit for HCC patients, it produces differential outcomes among HCC patients with different etiologies—for example, hepatitis C virus–related versus hepatitis B virus–related HCC—pointing to the difficulty of treating HCC [10]. Subsequently, additional targeted agents have been evaluated for HCC—for example, sunitinib, regorafenib, and brivanib—and have proven inferior to sorafenib [10]. Several new agents that have shown promise in phase II trials are still under evaluation. Among officially approved and well-tolerated pharmaceutical drugs, a first-generation antihistaminic drug, cyproheptadine, which is often used to treat allergies [11] and used as an appetite stimulant in cancer patients [12], has been demonstrated to have anticancer activity, including in mantle cell lymphoma, leukemia, and multiple myeloma [13,14]. Two independent post mortem case studies found the highest concentrations of cyproheptadine in bile and liver among different tissues and fluids, with liver-to-blood ratios ranging from 16.2 to 62.8 [15,16], indicating that cyproheptadine is favorably taken up by the liver. In addition, in an unexpected clinical finding, two advanced HCC patients with lung metastases achieved complete tumor remission upon treatment with a combination of cyproheptadine and thalidomide [17]. Taken together, these reports indicate a potent anti-HCC effect for cyproheptadine.

Although cyproheptadine has been shown to inhibit cancer cell growth by suppressing the PI3K/AKT signaling pathway, leading to down-regulation of D-cyclins and subsequently inducing apoptosis [18], the specific effects and mechanisms of action of cyproheptadine have not yet been identified in HCC. It would therefore be intriguing to explore the effects of this drug in HCC cell lines. Our present study investigated the effects of cyproheptadine on the growth of normal human hepatocytes and two HCC-derived cancer cell lines. The effects of this agent on cell cycle progression and apoptosis in HCC cells were also examined. Finally, we sought to reveal the underlying mechanisms involved in cell cycle arrest induced by cyproheptadine. Our results demonstrate that cyproheptadine induces cell cycle arrest in HepG2 cells through the induction of p38 MAPK, and in Huh-7 cells through the induction of p38 MAPK and CHK2, which mediate the induction of cell cycle regulatory proteins.

## Methods

### Ethics statement

The Ethics Committee of Ditmanson Medical Foundation Chia-Yi Christian Hospital approved this study.

### Preparation of cyproheptadine and cell cultures

Cyproheptadine hydrochloride, purchased from Sigma-Aldrich (St. Louis, MO), was dissolved in dimethyl sulfoxide (DMSO) at a concentration of 100 mM to provide stock solutions, which were then diluted with cell culture medium to desired concentrations ranging from 20 to 120 μM. Human HCC cell lines HepG2 and Huh-7 (Food Industry Research and Development Institute, Taiwan), as well as primary normal human hepatocytes (SC-5200, ScienCell Research Laboratories, Carlsbad, CA), were used as cell models. HepG2 and Huh-7 cells were cultured in Dulbecco’s modified Eagle’s medium supplemented with 10% fetal bovine serum (FBS), 100 units/ml penicillin, and 100 μg/ml streptomycin. Primary human hepatocytes were cultured in Hepatocyte Medium (ScienCell Research Laboratory, Carlsbad, CA) supplemented with 10% FBS, 100 units/ml penicillin, and 100 μg/ml streptomycin. All cell lines were cultured at 37°C under a humidified atmosphere containing 5% CO2.

### Cell viability assay

HepG2 and Huh-7 cells and primary human hepatocytes were seeded in 96-well plates at 1 × 10^4^ cells per well and cultured for 24 h. The cells were subsequently starved in culture medium without FBS for 24 h and then treated with cyproheptadine at various concentrations for 24 h. Cell viability was then determined by using Cell Counting Kit-8 (Sigma, Switzerland) according to the manufacturer’s protocol. In brief, the assay was performed with WST-8, which can be bio-reduced by cellular dehydrogenases to an orange formazan product that dissolves in cell culture medium. The production of formazan occurs only in living cells at a rate proportional to the number of living cells. After the cells were incubated with WST-8, the light absorbance of the culture medium in each well was measured at 450/655 nm on a Model 680 Microplate Reader (Bio-Rad, Hercules, CA). Cell viability was calculated relative to the untreated cells using the following equation:

$$ \mathrm{Viability}\kern0.2em \left(\%\right)=100\times \mathrm{Absorbance}\kern0.2em \mathrm{of}\kern0.2em \mathrm{treated}\kern0.2em \mathrm{group}\kern0.2em /\kern0.2em \mathrm{Absorbance}\kern0.2em \mathrm{of}\kern0.2em \mathrm{untreated}\kern0.2em \mathrm{group}. $$

A graph of cell viability versus concentration of the treatment agent was used to calculate the concentration that would return a cell viability of 50% (IC_50_). The selectivity index (SI), representing the cytotoxic selectivity of the agent against cancer cells relative to normal cells [19], was calculated from IC_50_ values as follows:

$$ \mathrm{S}\mathrm{I} = {\mathrm{IC}}_{50}\mathrm{of}\ \mathrm{the}\ \mathrm{given}\ \mathrm{agent}\ \mathrm{in}\ \mathrm{normal}\ \mathrm{cells}\ /\ {\mathrm{IC}}_{50}\mathrm{of}\ \mathrm{the}\ \mathrm{given}\ \mathrm{agent}\ \mathrm{in}\ \mathrm{cancer}\ \mathrm{cells}. $$

### Cell cycle analysis

HepG2 and Huh-7 were seeded in 6-well plates at 2 × 10^5^ cells per well and cultured for 24 h, starved in medium without FBS for 24 h, and then treated with 25–40 μM cyproheptadine for 48 h. Single-cell suspensions were prepared from the treated cells by trypsinization and resuspending in phosphate-buffered saline (PBS) and were then fixed with methanol at 4°C overnight. The fixed cells were rehydrated and washed twice with PBS before being stained by incubation with 5 μg/ml propidium iodide (Sigma, St. Gallen, Switzerland) and 1 mg/ml RNase A for 30 min in the dark at room temperature. The cells were then analyzed on a BD FACSCanto II flow cytometer (BD Biosciences, Franklin Lakes, NJ) with ModFit LT 3.3 as the data analysis software.

### Apoptosis detection

HCC cells were seeded on coverslips in 6-well plates at 2 × 10^5^ cells per well and cultured for 24 h, starved in medium without FBS for 24 h, and then treated with 40 μM cyproheptadine for either 24 h or 48 h. The treated cells, on coverslips, were gently washed with PBS and incubated with annexin V–FITC for 5 min in the dark at room temperature, followed by fixation in 2% formaldehyde. Subsequently, the coverslips were inverted on glass slides, and the cells were visualized using a fluorescence microscope (Olympus, Tokyo, Japan).

### Western blot analysis

HepG2 and Huh-7 were seeded in 6-well plates at 2 × 10^5^ cells per well and cultured for 24 h, starved in medium without FBS for 24 h, and then treated with 40 μM cyproheptadine for various durations. Total cellular proteins were extracted, and protein concentration was determined for the extracts using the Bio-Rad Protein Assay reagent (Bio-Rad) with bovine serum albumin as a standard. Each lysate (10 μg) was resolved on denaturing polyacrylamide gels and transferred electrophoretically to PVDF transfer membranes. After blocking with 3% blocker (Bio-Rad) in Tris-buffered saline with Tween 20 (TBST), the membranes were incubated at room temperature for 2 h with primary antibodies—1:5000 diluted antibody against GAPDH; 1:1000 diluted antibody against PARP, p21, p27, Rb (D20), phospho-Rb (Ser795), cyclin D1, p38 MAPK, phospho-p38 MAPK (Thr180/Tyr182), CHK2, phospho-CHK2 (Thr68), p53 (7F5), or phospho-p53 (Ser20) (Cell Signaling, Danvers, MA); or 1:1000 diluted antibody against p16^INK4A^ or HBP1 (Millipore, Temecula, CA). Immunoreactive proteins were detected by incubation with horseradish peroxidase–conjugated secondary antibodies for 1 h at room temperature. After washing with TBST, the reactive bands were developed with an enhanced chemiluminescent HRP substrate detection kit (Millipore, Billerica, MA) and identified using the BioSpectrum 800 imaging system (UVP).

### Statistical analysis

Data were expressed either as mean ± standard deviation (SD) or as a percentage relative to the untreated control. Differences between treated and untreated control groups were analyzed by one-way ANOVA followed by Dunnett’s test. Statistical significance was considered at a *P*-value <0.05 and at the 95% confidence level.

## Results

### Cyproheptadine treatment affects human HCC cell proliferation

**Figure 1:**
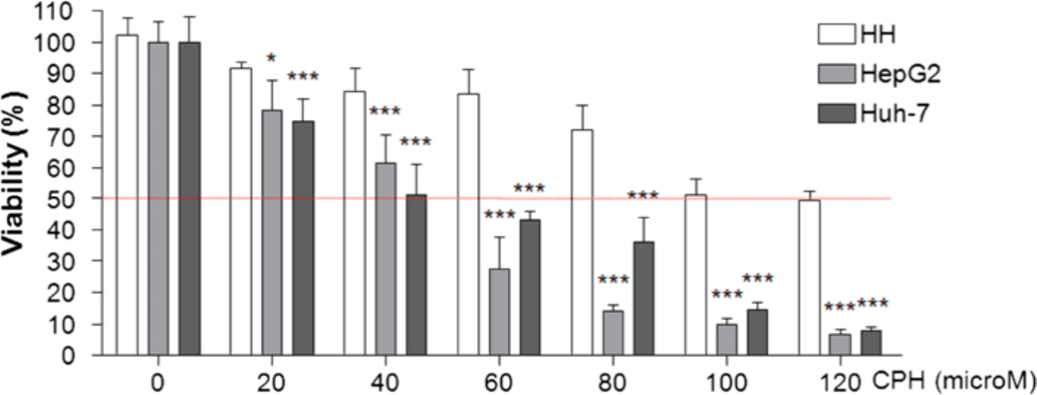
**Cytotoxicity of cyproheptadine toward normal human hepatocytes (HH) and HCC cell lines HepG2 and Huh-7.** Cells in 96-well plates were cultured for 24 h, starved in serum-free medium for 24 h, and then treated with various concentrations of cyproheptadine for 24 h. Viability was determined for the treated cells using Cell Counting Kit-8. Data are presented as mean ± SD (n = 6). Significant differences from the no-treatment control, determined by one-way ANOVA and Dunnett’s comparison test, are indicated by asterisks: *p < 0.05; ***p < 0.001.

We first performed an *in vitro* cell viability assay to compare the cytotoxicity of cyproheptadine in normal human hepatocytes and in HCC-derived human cancer cell lines. Analysis using Cell Counting Kit-8 revealed significant cytotoxicity of cyproheptadine to HepG2 and Huh-7 cells relative to normal hepatocytes at various concentrations and showed that cyproheptadine inhibited cell proliferation in a dose-dependent manner (Figure 1). A similar pattern was also observed in HepG2 and Huh-7 cells treated with cyproheptadine at a low-dosage range (0.5–5 μM) for 48 h (Additional file 1: Figure S1). The IC_50_ of cyproheptadine, determined as the concentration of the drug that inhibited cell growth by 50% after 24 h of treatment, was found to be 44.4, 44.7, and 118.1 μM in HepG2 cells, Huh-7 cells, and normal human hepatocytes, respectively. Cyproheptadine’s highly selective toxicity toward cancer cells is represented by its high selectivity index (SI) values for HepG2 and Huh-7 cells (2.7 and 2.6, respectively; Table 1).

**Table 1:** **Cytotoxic activities of cyproheptadine in HCC cell lines after 24 h of treatment**

IC_50_(µM)^a^			Selectivity index (SI^b^)	
HH^c^	HepG2	Huh-7	HepG2	Huh-7
118.1 ± 6.4	44.4 ± 6.1	44.7 ± 6.1	2.7	2.6

^a^Data are expressed as the means ± SD of ≥4 replicates.

^b^An SI value >2.6 indicates a high degree of cytotoxic selectivity.

^c^Normal human hepatocytes.

We previously reported the clinical finding that HCC patients achieved complete tumor remission upon treatment with a combination of cyproheptadine and thalidomide [17], which raises the possibility that thalidomide also has an inhibitory effect on HCC cells. Therefore, we used the same *in vitro* cell viability assay to measure the cytotoxicity mediated by thalidomide in HCC cells. Unexpectedly, thalidomide alone did not result in significant growth inhibition in either HepG2 or Huh-7 cells even when used at high dosage (200 μM) for 24 or 48 h (Additional file 1: Figure S2). These results indicate that thalidomide treatment alone is insufficient to inhibit the proliferation of HCC cells.

### Cyproheptadine arrests cell cycle progression in human HCC cells and induces apoptosis in Huh-7 cells

**Figure 2:**
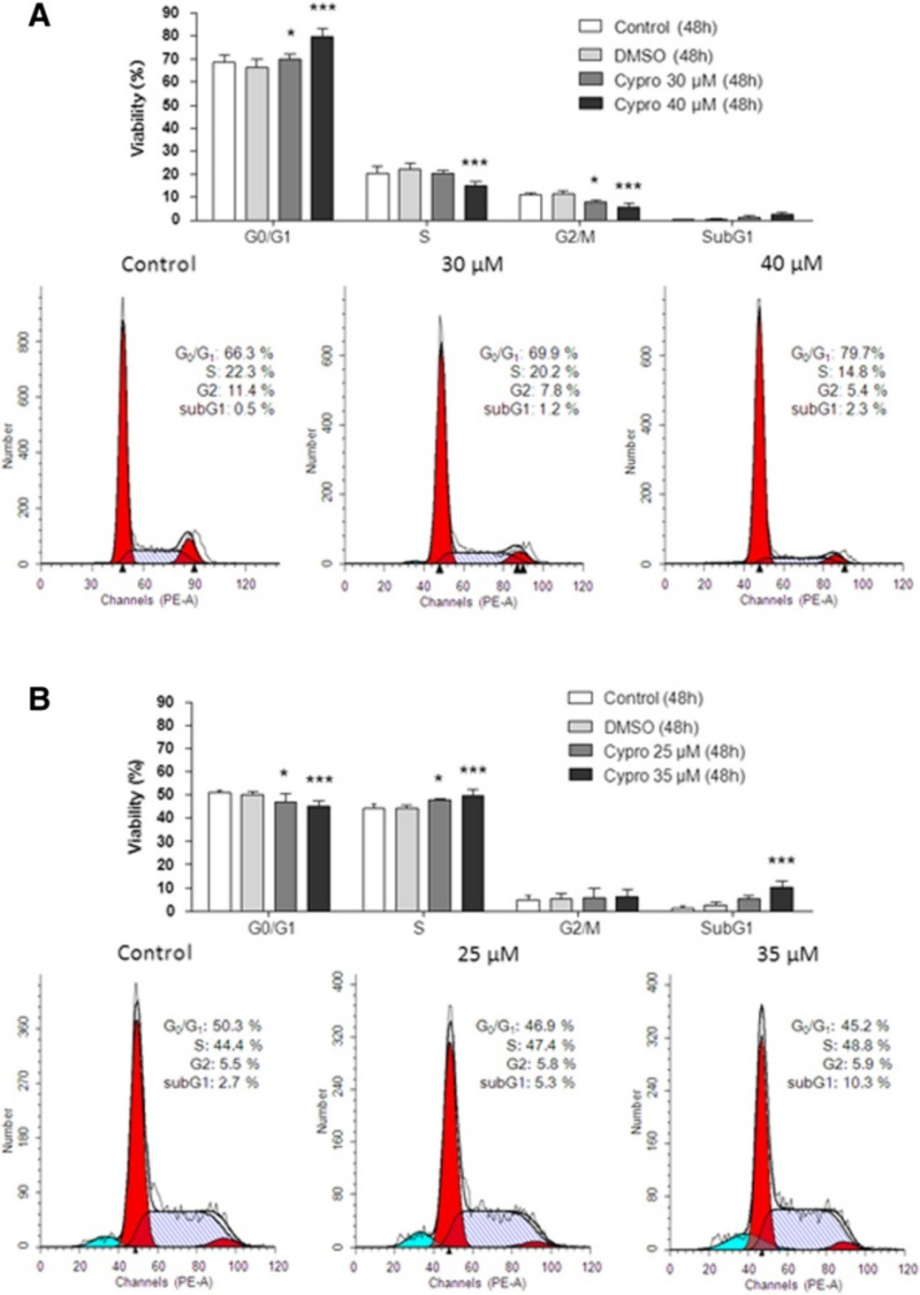
**Effects of cyproheptadine on the cell cycle in HCC cells.** HepG2 **(A)** and Huh-7 **(B)** cells in 6-well plates were cultured for 24 h, starved in serum-free medium for 24 h, and then treated with cyproheptadine at 30 or 40 μM (HepG2) or at 25 or 35 μM (Huh-7) for 48 h. Treated cells were stained with propidium iodide and analyzed by flow cytometry. Data are presented as mean ± SD (n = 4). Significant differences from the no-treatment control, determined by one-way ANOVA and Dunnett’s comparison test, are indicated by asterisks: *p < 0.05; ***p < 0.001. No difference was observed between the no-treatment control and the DMSO-only control in all test groups, indicating the absence of confounding effects from the DMSO solvent.

To explore the possible mechanisms through which cyproheptadine elicits its growth inhibitory effect, we determined if treatment with cyproheptadine hinders the cell cycle progression of HCC cells in concentration ranges close to the IC_50_ values. As shown by flow cytometry analysis, exposure to cyproheptadine at 30 and 40 μM for 48 h resulted in a significant increase in the percentage of HepG2 cells in the G0/G1 phase (*p* < 0.05 and *p* < 0.001, respectively; Figure. 2A) while decreasing the percentage in the G2/M phase and in both S and G2/M phases, respectively. In contrast, treatment with 25 and 35 μM cyproheptadine for 48 h significantly increased the percentage of Huh-7 cells in the S phase (*p* < 0.05 and *p* < 0.001, respectively; Figure 2B) and decreased the percentage in the G0/G1 phase (*p* < 0.05 and *p* < 0.001, respectively; Figure 2B).

**Figure 3:**
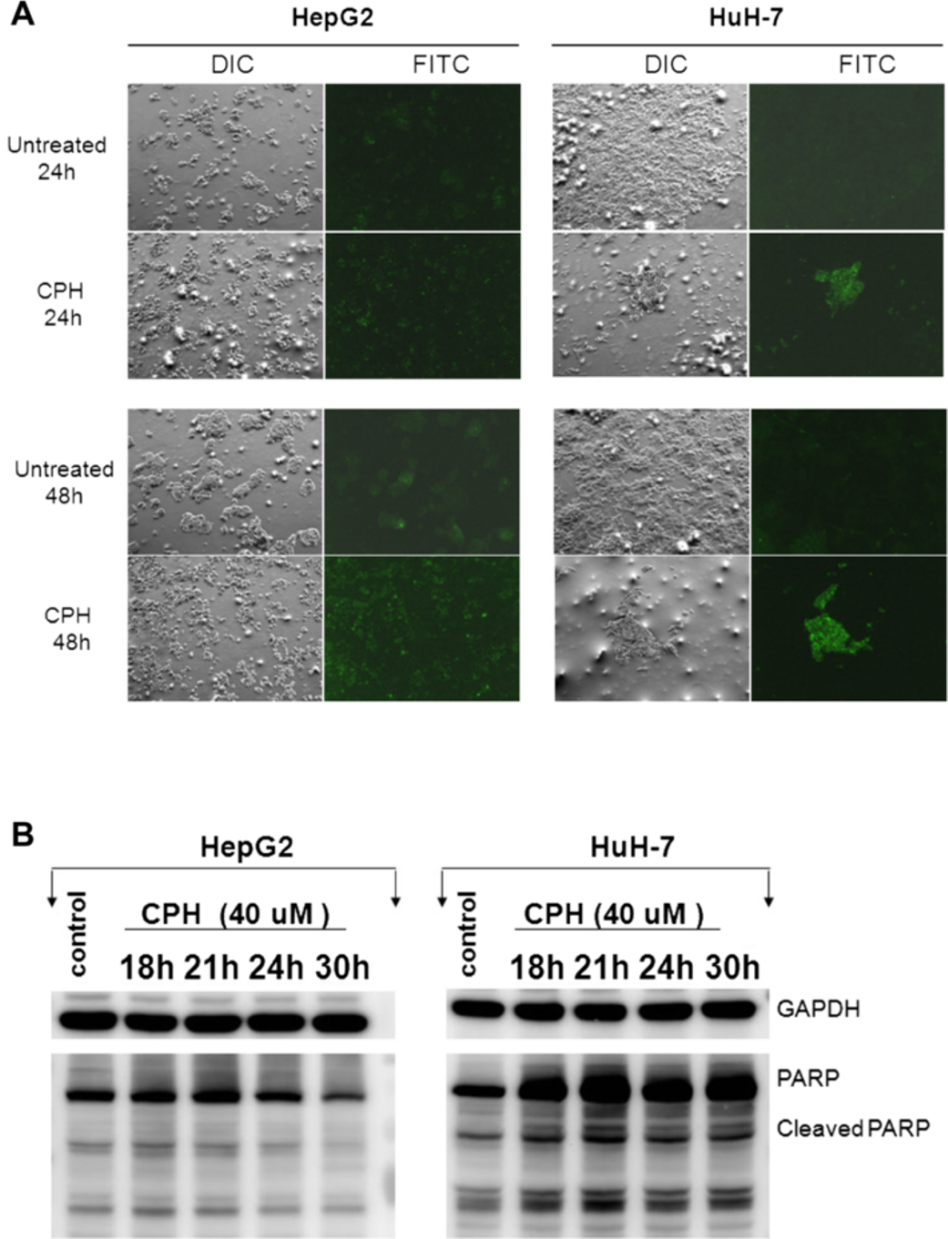
**Induction of apoptosis in Huh-7 cells by cyproheptadine. (A)** Annexin V staining assay. Cyproheptadine-treated HCC cells were stained with annexin V–FITC and analyzed by fluorescence microscopy. Untreated cells were primarily negative for annexin V staining, indicating that they were viable and not undergoing apoptosis. Treated cells undergoing apoptosis were observed to have positive annexin V staining. **(B)** Western blot analysis of PARP expression in cyproheptadine-treated HCC cells. The levels of PARP and its cleaved form increased significantly in Huh-7 cells after 18–30 h of treatment, but decreased in HepG2 cells after 24–30 h of treatment.

The above results suggest that cyproheptadine treatment leads to cell cycle arrest in HepG2 cells in the G1 phase and in Huh-7 cells at the G1/S transition. Accordingly, the increase in the proportion of HepG2 cells in G1 was significant at 40 μM of cyproheptadine (p < 0.001) and correlated with a reduction in the proportions in S and G2/M (p < 0.001) at this concentration. Similarly, the increase in the proportion of Huh-7 cells in the S phase was significant at 25 and 35 μM of cyproheptadine (*p* < 0.05 and *p* < 0.001, respectively) and correlated with a reduction in the proportion in G0/G1 at these concentrations (*p* < 0.05 and *p* < 0.001, respectively). We also observed that treatment with 35 μM cyproheptadine for 48 h produced a proportionately larger sub-G1 population in the treated Huh-7 cells relative to the untreated control (Figure 2B), indicating induction of cellular apoptosis. Therefore, we further investigated the effect of cyproheptadine treatment at 40 μM for different lengths of time (24 and 48 h) on the induction of apoptosis in HCC cells. As shown by annexin V–FITC binding analysis in Figure 3A (right panel set), cyproheptadine-treated Huh-7 cells were primarily positive for annexin V staining, indicating that they were undergoing apoptosis. However, significantly annexin V–FITC–positive cells were only sporadically observed in cyproheptadine-treated HepG2 cells (Figure 3A, left panel set), which is consistent with the result of flow cytometry analysis indicating the lack of a sub-G1 population in cyproheptadine-treated HepG2 cells (Figure 2A). Also, we assessed the effect of cyproheptadine treatment at 40 μM for different lengths of time (0, 18, 21, 24, and 30 h) on the induction of poly (ADP-ribose) polymerase (PARP) and its cleaved form, which is a hallmark of apoptosis, in HCC cells. As shown by western blot analysis (Figure 3B), the levels of PARP and its cleaved form increased significantly in Huh-7 cells following cyproheptadine treatment for 18–30 h, but decreased in HepG2 cells after 24–30 h of treatment. Together with the significantly increased sub-G1 population in the flow cytometry profile, these results indicate that cyproheptadine induces apoptosis in Huh-7 cells.

### Effects of cyproheptadine on cell cycle regulatory proteins

**Figure 4:**
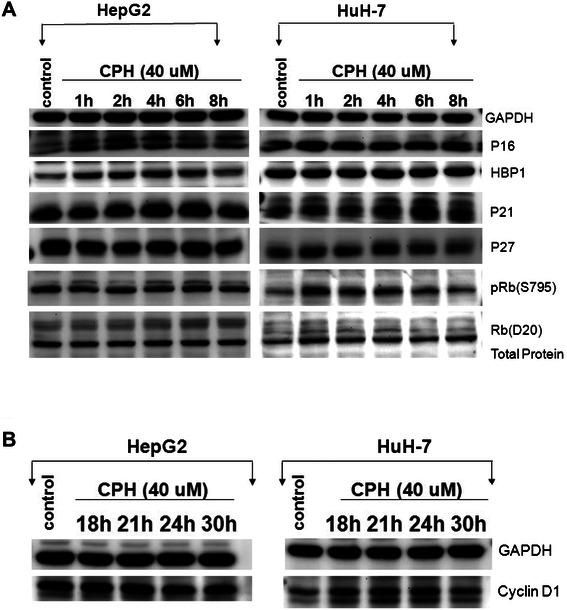
**Cyproheptadine alters the expression of cell cycle regulatory proteins. (A)** Western blot analysis of the expression of p16, HBP1, p21, p27, Rb, and phospho-Rb in HCC cells treated with 40 μM of cyproheptadine for different lengths of time. As shown in the figure, the levels of p16 and HBP1 increased in HepG2 cells after treatment with cyproheptadine for 1–4 h, followed by a gradual decrease during 6–8 h, but did not change significantly in Huh-7 cells. The levels of p21 and p27 increased in Huh-7 cells after 4–6 h and 1–4 h of treatment, respectively, but did not change significantly in HepG2 cells. The level of phospho-Ser795 Rb decreased in a time-dependent manner after 2–8 h of treatment in Huh-7 cells, but not in HepG2 cells. **(B)** Western blot analysis of cyclin D1 expression in cyproheptadine-treated HCC cells. The result shows a moderate decrease in the cyclin D1 level in Huh-7 cells after treatment with cyproheptadine for 30 h, but not in HepG2 cells.

To elucidate the molecular mechanisms by which cyproheptadine induces cell cycle arrest, we examined the expression of several cell cycle regulatory proteins. HCC cells were treated with 40 μM cyproheptadine for different lengths of time and analyzed by western blotting. The results show that the expression of p16^INK4A^ increased significantly in HepG2 cells following treatment with cyproheptadine for 1–4 h (Figure 4A, left panel set) but did not change significantly in Huh-7 cells (Figure 4A, right panel set). It has been shown recently that the transcription factor HMG box-containing protein 1 (HBP1) targets p16^INK4A^ through direct sequence-specific binding to its promoter and up-regulates its expression [20]. We were thus interested in determining whether the expression profile of HBP1 correlates with that of p16^INK4A^ in our HCC cell lines. Western blot analysis showed that the expression of HBP1 increased significantly in HepG2 cells following treatment with cyproheptadine for 1–4 h, which matched the pattern of change in p16^INK4A^ expression in this cell line (Figure 4A, left panel set). In contrast, no significant changes in the level of HBP1 were observed in Huh-7 cells, in keeping with the expression pattern of p16^INK4A^ in this cell line (Figure 4A, right panel set).

Next, we analyzed the effect of cyproheptadine on the expression of the cyclin-dependent kinase inhibitors p21 and p27 in HCC cells. As detected by western blotting, the levels of p21 and p27 increased significantly in Huh-7 cells following treatment with cyproheptadine for 1–6 h and 1–4 h, respectively (Figure 4A, right panel set), but no significant changes in these proteins were observed in HepG2 cells (Figure 4A, left panel set). We also analyzed the effect of cyproheptadine on retinoblastoma protein (Rb) phosphorylation and found a strong time-dependent decrease in the level of phospho-Ser795 Rb in Huh-7 cells but not in HepG2 cells (Figure 4A). In addition, we examined the effect of cyproheptadine on the expression of cyclin D1. Although the level of cyclin D1 did not change in response to cyproheptadine treatment in HepG2 cells (Figure 4B, left panel set), a moderate decrease in cyclin D1 expression was observed in Huh-7 cells after 30 h of treatment (Figure 4B, right panel set).

### Cyproheptadine-induced cell cycle arrest involves p38 MAPK activation in HepG2 cells and involves both p38 MAPK and CHK2 activation in Huh-7 cells

**Figure 5:**
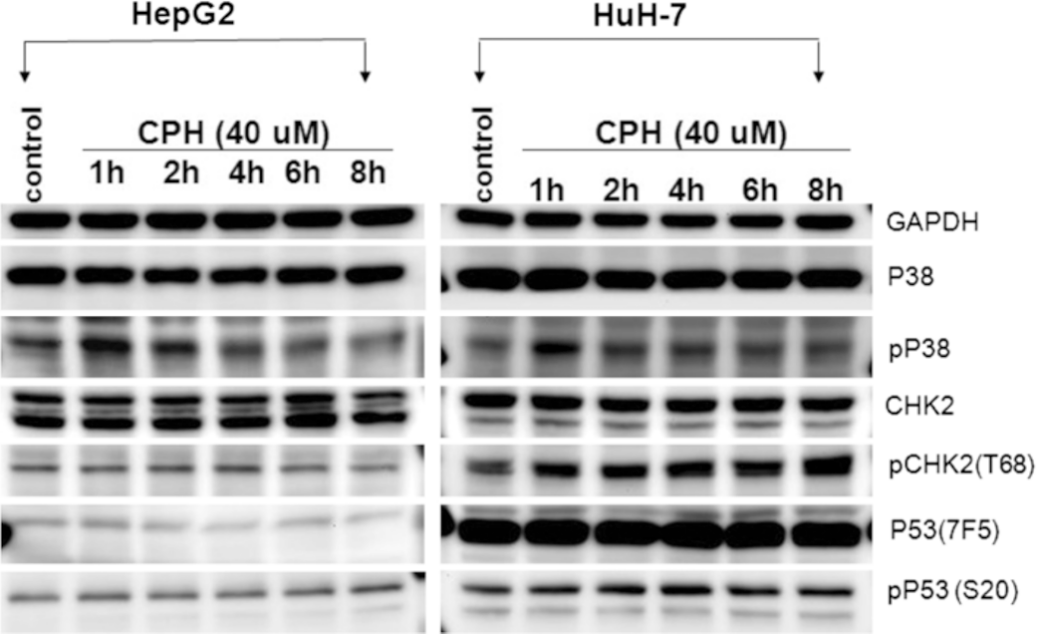
**Cyproheptadine induces p38 MAPK activation in HepG2 cells and activation of p38 MAPK and CHK2 in Huh-7 cells.** Western blot analysis was performed to detect p38, CHK2, p53, and their phosphorylated forms in HCC cells following treatment with 40 μM cyproheptadine for different lengths of time. The level of Thr180/Tyr182-phosphorylated p38 MAPK markedly increased in both HCC cell lines after 1 h of treatment, indicating p38 MAPK activation. The level of Thr68-phosphorylated CHK2 increased independently of the level of Ser20-phosphorylated p53 in Huh-7 cells (but not in HepG2 cells) after treatment, indicating CHK2’s p53-independent role in cell cycle arrest in this cell line.

Previous studies have demonstrated that p38 MAPK plays a role in cell cycle regulation by activating the cell cycle checkpoints at G2/M and at G1/S in response to cellular stress [21,22]. To determine whether the activation of p38 MAPK is involved in cyproheptadine-induced cell cycle arrest, we examined the induction of Thr180/Tyr182-phosphorylated p38 MAPK in cyproheptadine-treated HCC cells. Following treatment with 40 μM cyproheptadine for different lengths of time, cell lysates were prepared and analyzed by western blotting using antibodies specific for p38 MAPK and Thr180/Tyr182-phosphorylated p38 MAPK. As shown in Figure 5, a significant increase in p38 MAPK activation occurred in both HCC cell lines after treatment for 1 h, as indicated by the increased levels of Thr180/Tyr182-phosphorylated p38 MAPK. The total amount of p38 MAPK was unaffected by cyproheptadine treatment in both cell lines (Figure 5). To validate the role of p38 MAPK in cyproheptadine’s effects, SB202190, an inhibitor of p38, was used to assess the effect of p38 inhibition on cyproheptadine-induced p38 MAPK activation and expression of cell cycle–regulating proteins including HBP1, p16^INK4A^, p21, and p27. We found that, in contrast to the increased p38 MAPK phosphorylation and expression of cell cycle–regulating proteins upon cyproheptadine treatment, co-treatment with SB202190 and cyproheptadine significantly inhibited p38 MAPK phosphorylation in both HCC cell lines, decreased HBP1 and p16^INK4A^ expression in HepG2 cells, and decreased p27 expression in Huh-7 cells (Additional file 1: Figure S3). These results thus correlate cyproheptadine-mediated increase in p38 MAPK phosphorylation with an immediate increase in HBP1 and p16^INK4A^ expression in HepG2 cells and with a subsequent increase in p27 expression in Huh-7 cells.

CHK2 has been found to be dispensable for p53-mediated cell cycle arrest [23,24]. We were interested in exploring a p53-independent role for CHK2 in inducing cell cycle arrest because the tumor suppressor p53 is frequently mutated in cancer cells and the Huh-7 HCC cell line used in this study is p53 defective [25]. Using antibodies specific for CHK2, Thr68-phosphorylated CHK2, p53, and Ser20-phosphorylated p53, we detected a time-dependent increase in the level of Thr68-phosphorylated CHK2 and no change in the level of total CHK2 in Huh-7 cells (Figure 5, right panel set). In contrast, no significant changes in the levels of phospho-Thr68 CHK2 and total CHK2 were observed in HepG2 cells (Figure 5, left panel set). Furthermore, no significant increase in p53 activation occurred in either HCC cell line following cyproheptadine treatment, as indicated by the absence of significant changes in the level of Thr20-phosphorylated p53. Accordingly, the amount of total p53 was also unaffected by cyproheptadine treatment in both cell lines (Figure 5). These results suggest that cyproheptadine is able to induce CHK2 activation in p53-defective HCC cells to cause cell cycle arrest.

## Discussion

Inadequate outcomes in the treatment of HCC have necessitated the development of alternative approaches to chemotherapy. Recently, an H1 histamine receptor antagonist and serotonin receptor blocker, cyproheptadine, has been reported for its anticancer activity, which resulted in the induction of cancer cell apoptosis in mantle cell lymphoma, leukemia, and multiple myeloma [13,14] and complete remission in two advanced HCC patients with lung metastases upon treatment with a combination of cyproheptadine and thalidomide [17]. Notably, despite its anti-angiogenic effects, thalidomide alone is insufficient treatment [26,27] and must be combined with other drugs or therapies in the treatment of cancer [28,29]. In addition, previous *in vitro* studies on human prostate carcinoma cells [30], human glioma cells [31], and Ehrlich ascites tumor cells [32] support the notion that thalidomide is not cytotoxic to cancer cells, indicating that the growth inhibition effect of thalidomide depends not only on the dosage of the drug but also on the cell type [33]. Consistently, we have demonstrated through our *in vitro* analysis that thalidomide treatment alone is not beneficial in terms of cellular cytotoxicity toward HCC cells (Additional file 1: Figure S2). In view of these results, cyproheptadine represents an attractive anticancer drug candidate, especially as it is already in clinical use as an antihistamine and appetite stimulant and is well tolerated and officially approved for years.

It is not known, however, whether antitumor concentrations of cyproheptadine are achievable in the human body. Daily treatment with cyproheptadine could produce serum levels of the drug higher than those observed after a single dose because of the slow elimination of cyproheptadine, which has a plasma half-life of metabolites of about 16 h [34]. Moreover, in a patient who overdosed on cyproheptadine and ethanol, tissue concentrations of cyproheptadine exceeded serum concentrations by a factor of up to 3 to 16 [16], indicating large-volume, extensive distribution of cyproheptadine into tissues [35]; the concentration of cyproheptadine in bile has been observed to reach as high as 30.7 mg/L (106.8 μM) [15], which is more than twice the concentration required to produce an antitumor effect in our *in vitro* study. Therefore, antitumor concentrations of cyproheptadine in human tissues might be attainable with daily high-dose treatment.

In the present study, we report the *in vitro* antiproliferative effects of cyproheptadine in HepG2 (p53^wt/wt^, or p53-wild-type) and Huh-7 (p53^del/mut^, or p53-defective) HCC cells. The results clearly demonstrate that cyproheptadine has similar cytotoxic effects in both HCC cell lines despite their different p53 genetic backgrounds. Furthermore, since an SI value <2 indicates general toxicity of the agent [36], the SI values we determined for cyproheptadine (Table 1) reveal a high degree of cytotoxic selectivity toward HCC cells and entail greatly reduced adverse side effects associated with normal hepatocytes. Importantly, the high SI values of cyproheptadine make it a good candidate for an anticancer agent. The high cytotoxic selectivity of cyproheptadine should be further investigated.

Our cell cycle analysis revealed that cyproheptadine leads to cell cycle arrest in HepG2 in the G1 phase while arresting the cell cycle progression of Huh-7 cells at the G1/S transition (Figure 2A and B). To elucidate cyproheptadine’s differential effects on the cell cycle in these cells, we examined the expression status of various cell cycle mediators. We were able to correlate cyproheptadine-induced G1 arrest in HepG2 cells with the induction of p16^INK4A^, which is known to inhibit the activation of cyclin-dependent protein kinase Cdk4/6 [37]. Importantly, we show for the first time the concurrent induction of HBP1 and p16^INK4A^ expression by cyproheptadine, and this parallel induction suggests that a common signaling event engages HBP1 and p16^INK4A^ expression. However, we cannot exclude the simultaneous effect of HBP1, a transcription factor, promoting the expression of p16^INK4A^ because the p16^INK4A^ gene has been described as a novel target of transcription regulation by HBP1 [20]. As for cyproheptadine-induced G1/S arrest in Huh-7 cells, a different set of regulatory proteins may be involved. We show that cyproheptadine treatment induces the expression of p21 and p27 in Huh-7 cells (Figure 4A, right panel set). Because Huh-7 cells contain a defective mutation in the p53 gene, this p21 and p27 induction is independent of p53, as evidenced by an unchanged level of Ser20-phosphorylated p53 with and without treatment (Figure 5, right panel set). Consistently, p53-independent induction of p21 and p27 expression has been reported previously [38-40]. We also observed a significant time-dependent decrease in the hyperphosphorylated form of Rb in cyproheptadine-treated Huh-7 cells (Figure 4A, right panel set). Therefore, it is likely that the cyproheptadine-mediated induction of p21 and p27 expression contributes to the suppression of the kinase activity of the CDK2–cyclin E complex [41]. As a consequence, Rb remains in a hypophosphorylated state, leading to cell cycle arrest at the G1/S transition [42-44].

**Figure 6:**
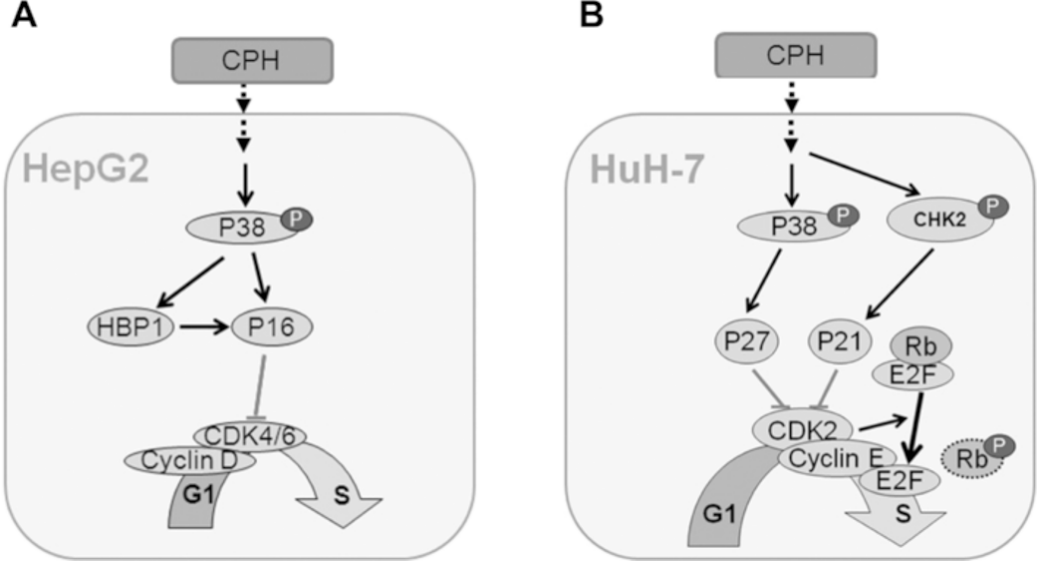
**Schematic diagram of proposed effects of cyproheptadine on the cell cycle in HCC cells. (A)** In HepG2 cells, cyproheptadine treatment causes significant activation of p38 MAPK activity, which subsequently mediates induction of p16^INK4A^ and HBP1. As a target of transcriptional regulation by HBP1, p16^INK4A^ gene expression can be further promoted. Cytosolic p16^INK4A^ may ultimately inhibit the activation of the cyclin-dependent protein kinase Cdk4/6, leading to cell cycle arrest in the G1 phase. **(B)** In Huh-7 cells, cyproheptadine treatment causes significant activation of p38 MAPK activity, which subsequently mediates the p53-independent induction of p27. At the same time, phosphorylation-activated CHK2 can promote p21 induction in a p53-independent way. The induced p21 and p27 contribute to a reduction in the kinase activity of the CDK2–cyclin E complex, which causes Rb to remain in a hypophosphorylated state, leading to cell cycle arrest at the G1/S transition.

We initially found that in response to cyproheptadine, p38 MAPK was rapidly and transiently activated in both HCC cell lines, as seen from the increased level of Thr180/Tyr182-phosphorylated p38 MAPK following treatment with the drug (Figure 5; Additional file 1: Figure S3). This result prompted our western blot analysis of cyproheptadine’s effects on cell cycle–regulating proteins, including HBP1, p16^INK4A^, p21, and p27. The MAPK superfamily is known to play an important role in multiple cellular activities including proliferation, growth inhibition, differentiation, and apoptotic responses to a variety of extracellular stimuli [45-47]. Previous studies have demonstrated the involvement of p38 MAPK signaling in the regulation of cell cycle progression, especially at the G1/S phase [48,49]. Our results show that the cyproheptadine-mediated increase in p38 MAPK phosphorylation is followed by an immediate increase in HBP1 and p16^INK4A^ expression in HepG2 cells and a subsequent increase in p27 expression in Huh-7 cells (Figure 4; Additional file 1: Figure S3). These data suggest that activation of p38 MAPK signaling may serve as a common pathway by which cyproheptadine up-regulates HBP1, p16^INK4A^, and p27. Accordingly, it has been reported that p38 MAPK can mediate HBP1 phosphorylation and thereby increase the stability and the protein level of HBP1 [50], and that p38 MAPK can facilitate G1/S arrest by up-regulating p16^INK4A^ expression [51]. Our findings are also consistent with those of Kim *et al*. [52] and Mukhopadhyay *et al.* [41], who demonstrated that p38 MAPK activation leads to the induction of p21 and p27 expression in prostate cancer cells. As CHK2 activation has been found to be responsible for the induction of p21 expression in p53-deficient SK-BR-3 breast cancer cells and HaCaT immortalized keratinocytes [24], we were interested in determining whether CHK2 is activated in cyproheptadine-treated Huh-7 cells in which p21 expression is up-regulated. Our result shows for the first time that CHK2 is rapidly and increasingly activated in Huh-7 cells in response to cyproheptadine, as demonstrated by a time-dependent increase in the level of Thr68-phosphorylated CHK2 (Figure 5, right panel set). This result also reveals a p53-independent role for CHK2 in p21 induction that may contribute to tumor suppression and the outcome of cyproheptadine treatment. Furthermore, it has been reported that cyproheptadine altered cyclin D1 expression in myeloma and leukemia [13]. In our study, although cyproheptadine did not alter cyclin D1 expression in HepG2 cells, it did induce a moderate decrease in cyclin D1 expression in Huh-7 cells following treatment for 30 h (Figure 4B). It is likely that p38 MAPK can negatively regulate cyclin D1 at the level of transcription [53] or directly phosphorylate cyclin D1, leading to cyclin D1 ubiquitination and degradation [54]. Nevertheless, we show that the impact of cyproheptadine on cell cycle regulatory proteins is mediated through the activation of p38 MAPK activity. On the basis of our collective data, we present a schematic summary of the hypothesized effects of cyproheptadine on the cell cycle in the two HCC cell lines in Figure 6.

More than a few studies have reported that cell cycle arrest may lead to the induction of apoptosis [55,56]. Therefore, we were interested to see whether cyproheptadine could induce apoptosis in HCC cells. We observed that Huh-7 cells underwent cyproheptadine-induced apoptosis, as evidenced by the presence of a sub-G1 population (Figure 2B), positive annexin V staining (Figure 3A), and an increased level of PARP and its cleavage product (Figure 3B) in the treated cells, indicating that cyproheptadine is a potent inducer of apoptosis. However, flow cytometry, annexin V–FITC apoptosis detection, and biochemical analyses revealed no significant increases in apoptosis in HepG2 cells after cyproheptadine treatment. This may be due to apoptotic pathway deficiencies [57] in this cancer cell line, in which the kinase, receptor-interacting protein 1 (RIP1), likely targets the mitochondria, leading to surplus formation of reactive oxygen species [58] and the subsequent induction of necrotic cell death [59-61]. In addition, cyproheptadine-induced activation of p38 MAPK signaling and the resulting induction of HBP1 and p16^INK4A^ expression may trigger premature senescence [20], which has been suggested to play a role in tumor suppression by reducing the replicative potential of cells [62] and is frequently detected in tumors obtained from patients who had undergone genotoxic chemotherapy [63].

## Conclusions

In conclusion, the findings presented in this report demonstrate the anticancer potential of cyproheptadine *in vitro* in two human hepatocellular cancer cell lines, HepG2 and Huh-7. A high degree of selectivity for cancer cells, relatively low toxicity to normal hepatocytes, and a possible liver sequestration of the drug make cyproheptadine a good drug candidate for liver cancer chemotherapy; however, animal studies will be required to further validate these results *in vivo*. We have also demonstrated that cyproheptadine interferes with cell cycle progression via the activation of p38 MAPK activity in HepG2 cells and the activation of both p38 MAPK and CHK2 activities in Huh-7 cells, which subsequently mediate, through G1/S cell cycle regulatory proteins, the induction of apoptotic or non-apoptotic cell death in these liver cancer cells. Thus, we have revealed a novel cellular target for an interesting antihistamine drug whose effects encompass a wide range of biological events including growth inhibition and cell death induction. The results from our present study provide a strong foundation for further development of cyproheptadine as a novel treatment option that, in combination with other compounds, may be useful for human hepatocellular cancer prevention and therapy.

## Additional file

Additional file 1: Figure S1. Cytotoxicity of low-dose cyproheptadine toward HepG2 (A) and Huh-7 cells (B). Cells were treated with various concentrations of cyproheptadine for 24 h or 48 h. Cell viability data are presented as mean ± SD (n = 6). Significant differences from the no-treatment control, determined by one-way ANOVA and Dunnett’s comparison test, are indicated as *p < 0.05; **p < 0.01; ***p < 0.001. **Figure S2** Cytotoxicity of thalidomide toward HepG2 and Huh-7 cells. Cells were treated with various concentrations of thalidomide for 24 h (A) or 48 h (B). Cell viability data are presented as mean ± SD (n = 6). No significant differences from the no-treatment control were determined. **Figure S3** Cyproheptadine induces p38 MAPK activation to mediate the expression of cell cycle regulatory proteins in HCC cells. HepG2 (A) and Huh-7 cells (B) were treated with 40 μM cyproheptadine (column 2), with 10 μM of the p38 MAPK inhibitor SB202190 (column 4), or with a combination of 40 μM cyproheptadine and 10 μM SB202190 (column 3) for different lengths of time. Expression of Thr180/Tyr182-phosphorylated p38 MAPK, p16, HBP1, p21, and p27 were analyzed by western blotting. A no-treatment control was also included (column 1). The level of Thr180/Tyr182-phosphorylated p38 MAPK increased in both HCC cell lines after treatment with cyproheptadine for 1–4 h (A and B, column 2). The increase in phospho-p38 MAPK was significantly less after co-treatment with cyproheptadine and SB202190 (A and B, column 3). In HepG2 cells, the levels of p16 and HBP1 increased after treatment with cyproheptadine for 1–4 h (A, column 2) but decreased after co-treatment with cyproheptadine and SB202190 (A, column 3). In Huh-7 cells, both p21 and p27 increased in level after 1–2 h of treatment with cyproheptadine, whereas only p27 decreased after co-treatment with cyproheptadine and SB202190 (B, column 3).
