# Supplementary material for: Cyproheptadine, an antihistaminic drug, inhibits proliferation of hepatocellular carcinoma cells by blocking cell cycle progression through the activation of P38 MAP kinase
Source: BMC Cancer. 2015 Mar 17;15:134. doi: 10.1186/s12885-015-1137-9 (PMC4383201; doi:10.1186/s12885-015-1137-9)
Supplement: Additional file 1: Figure S1. — Cytotoxicity of low-dose cyproheptadine toward HepG2 (A) and Huh-7 cells (B). Cells were treated with various concentrations of cyproheptadine for 24 h or 48 h. Cell viability data are presented as mean ± SD (n = 6). Significant differences from the no-treatment control, determined by one-way ANOVA and Dunnett’s comparison test, are indicated as *p < 0.05; **p < 0.01; ***p < 0.001. Figure S2 Cytotoxicity of thalidomide toward HepG2 and Huh-7 cells. Cells were treated with various concentrations of thalidomide for 24 h (A) or 48 h (B). Cell viability data are presented as mean ± SD (n = 6). No significant differences from the no-treatment control were determined. Figure S3 Cyproheptadine induces p38 MAPK activation to mediate the expression of cell cycle regulatory proteins in HCC cells. HepG2 (A) and Huh-7 cells (B) were treated with 40 μM cyproheptadine (column 2), with 10 μM of the p38 MAPK inhibitor SB202190 (column 4), or with a combination of 40 μM cyproheptadine and 10 μM SB202190 (column 3) for different lengths of time. Expression of Thr180/Tyr182-phosphorylated p38 MAPK, p16, HBP1, p21, and p27 were analyzed by western blotting. A no-treatment control was also included (column 1). The level of Thr180/Tyr182-phosphorylated p38 MAPK increased in both HCC cell lines after treatment with cyproheptadine for 1–4 h (A and B, column 2). The increase in phospho-p38 MAPK was significantly less after co-treatment with cyproheptadine and SB202190 (A and B, column 3). In HepG2 cells, the levels of p16 and HBP1 increased after treatment with cyproheptadine for 1–4 h (A, column 2) but decreased after co-treatment with cyproheptadine and SB202190 (A, column 3). In Huh-7 cells, both p21 and p27 increased in level after 1–2 h of treatment with cyproheptadine, whereas only p27 decreased after co-treatment with cyproheptadine and SB202190 (B, column 3). [file 12885_2015_1137_MOESM1_ESM.docx]

**Supplemental Information**

**Cyproheptadine, an Antihistaminic Drug, Inhibits Proliferation of Hepatocellular Carcinoma Cells by Blocking Cell Cycle Progression through the Activation of P38 MAP Kinase**

Yu-Min Feng, Chin-Wen Feng, Syue-Yi Chen, Hsiao-Yen Hsieh, Yu-Hsin Chen, Cheng-Da Hsu


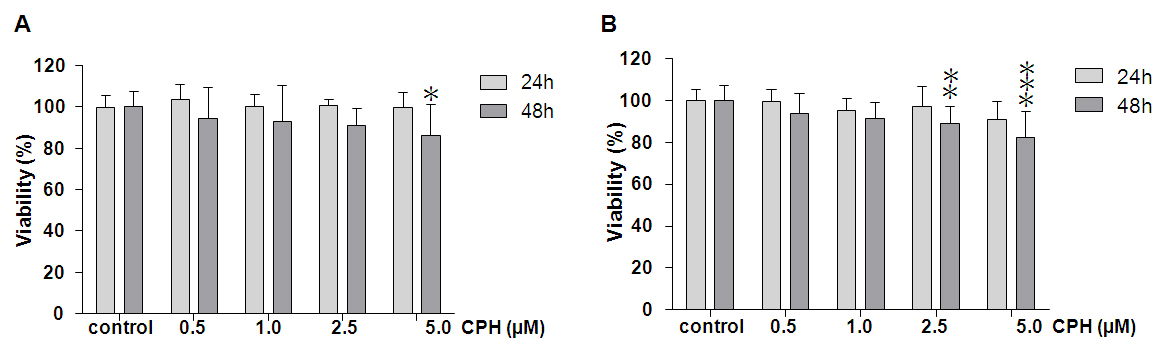


**Figure S1 Cytotoxicity of low-dose cyproheptadine toward HCC cell lines HepG2 (A) and Huh-7 (B).**

Cells in 96-well plates were cultured for 24 h, starved in serum-free medium for 24 h, and then treated with various concentrations of cyproheptadine for 24 h or 48 h. The viability of the treated cells was assessed using Cell Counting Kit-8. Data are presented as mean ± SD (n = 6). Significant differences from the no-treatment control, determined by one-way ANOVA and Dunnett’s comparison test, are indicated by asterisks: *p < 0.05; **p < 0.01; ***p < 0.001.


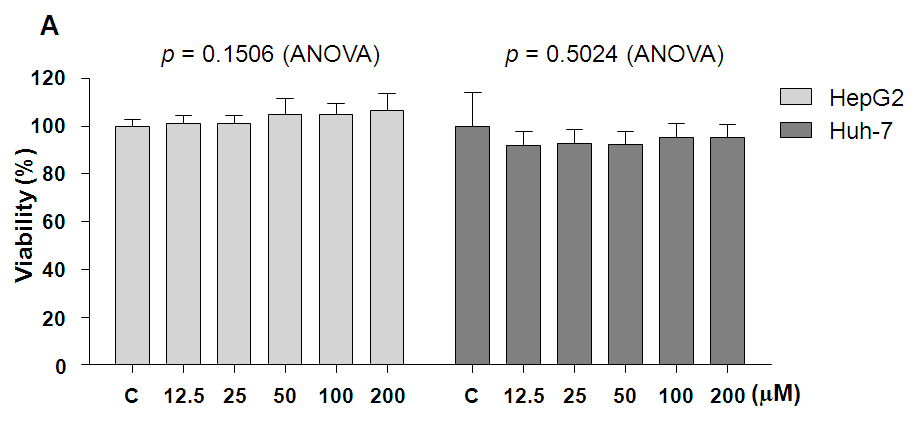


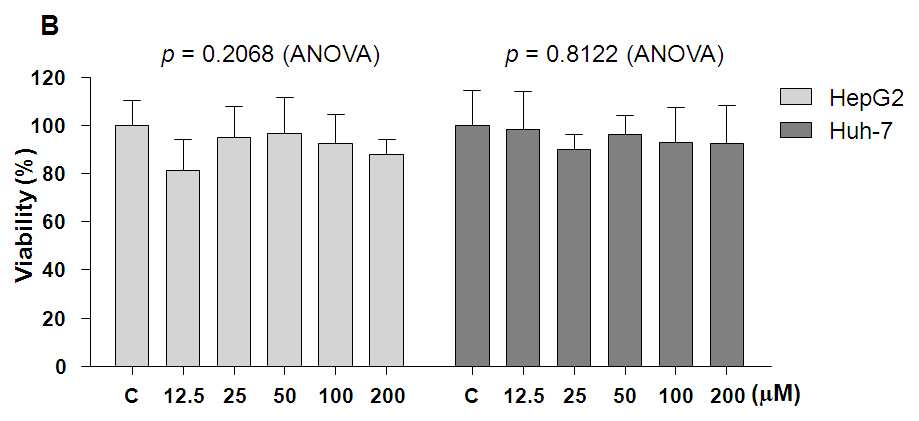


**Figure S2 Cytotoxicity of thalidomide toward HCC cell lines HepG2 and Huh-7.**

Cells in 96-well plates were cultured for 24 h, starved in serum-free medium for 24 h, and then treated with various concentrations of thalidomide for 24 h (A) or 48 h (B). The viability of the treated cells was assessed using Cell Counting Kit-8. Data are presented as mean ± SD (n = 6). No significant differences from the no-treatment control were determined by one-way ANOVA and Dunnett’s comparison test.


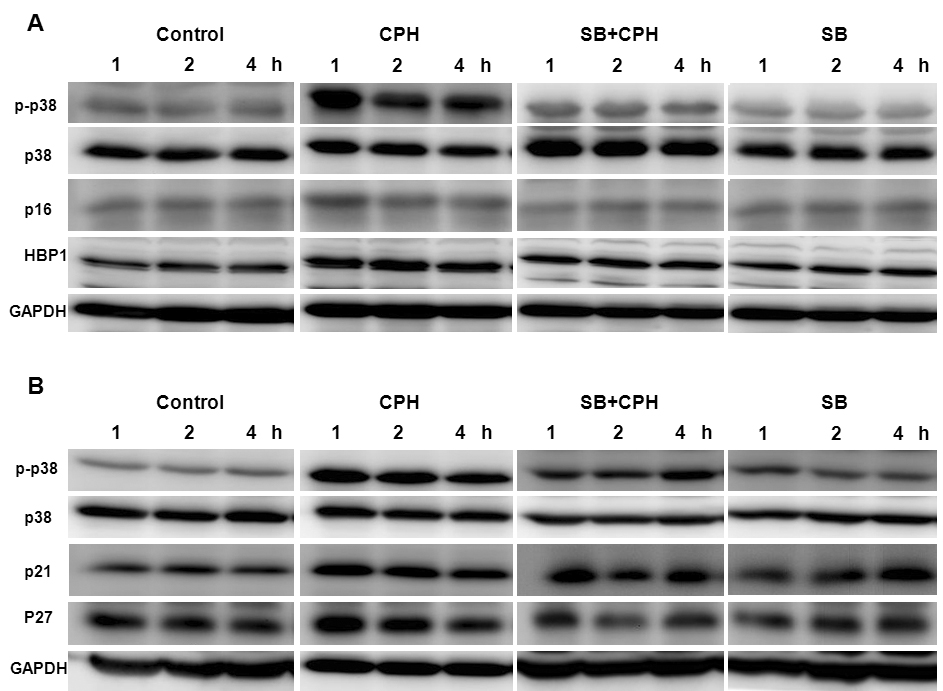


**Figure S3 Cyproheptadine induces p38 MAPK activation to mediate the expression of cell cycle regulatory proteins in HCC cells.**

HepG2 (A) and Huh-7 cells (B) were treated with 40 µM cyproheptadine (column 2), with 10 µM of the p38 MAPK inhibitor SB202190 (column 4), or with a combination of 40 µM cyproheptadine and 10 µM SB202190 (column 3) for different lengths of time. Treated cells were analyzed by western blotting for p38 MAPK activation and the expression of p16, HBP1, p21, and p27. A no-treatment control was also included (column 1). As shown in the figure, the level of Thr180/Tyr182-phosphorylated p38 MAPK markedly increased in both HCC cell lines after treatment with cyproheptadine for 1–4 h (A and B, column 2), indicating p38 MAPK activation. The increase in phospho-p38 MAPK was significantly less after co-treatment with cyproheptadine and SB202190 (A and B, column 3). In HepG2 cells, the levels of p16 and HBP1 increased after treatment with cyproheptadine for 1–4 h (A, column 2) but decreased after co-treatment with cyproheptadine and SB202190 (A, column 3). In Huh-7 cells, both p21 and p27 increased in level after 1–2 h of treatment with cyproheptadine, whereas only p27 decreased after co-treatment with cyproheptadine and SB202190 (B, column 3).

**Supplemental Experimental Procedures**

**Preparation of thalidomide and cell cultures**

Thalidomide, purchased from Sigma-Aldrich (St. Louis, MO), was dissolved in dimethyl sulfoxide at a concentration of 500 mM to provide stock solutions, which were then diluted with cell culture medium to desired concentrations ranging from 12.5 to 200 µM. Human HCC cell lines HepG2 and Huh-7 (Food Industry Research and Development Institute, Taiwan), used as cell models, were cultured in Dulbecco's modiﬁed Eagle's medium supplemented with 10% fetal bovine serum (FBS), 100 units/ml penicillin, and 100 μg/ml streptomycin. All cell lines were cultured at 37°C under a humidified atmosphere containing 5% CO_2_.

**Cell viability assay**

HepG2 and Huh-7 cells were seeded in 96-well plates at 1 × 10^4^ cells per well and cultured for 24 h. The cells were subsequently starved in culture medium without FBS for 24 h and then treated with cyproheptadine or thalidomide at various concentrations for various durations. Cell viability was then determined by using Cell Counting Kit-8 (Sigma, Switzerland) according to the manufacturer's protocol. In this assay, the reagent WST-8 was bio-reduced by cellular dehydrogenases, at a rate proportional to the number of living cells present, to a soluble formazan product, whose concentration in the culture medium was measured by absorbance at 450/655 nm on a Model 680 Microplate Reader (Bio-Rad, Hercules, CA). Cell viability was calculated relative to the control cells using the following equation:

Viability (%) = 100 × Absorbance of treated group / Absorbance of untreated group.

**Western blot analysis**

HepG2 and Huh-7 were seeded in 6-well plates at 2 × 10^5^ cells per well and cultured for 24 h, starved in medium without FBS for 24 h, and then treated with 40 µM cyproheptadine or 10 µM of the p38 inhibitor SB202190 for various durations. For co-treatment analysis, starved HCC cells were pretreated with 10 µM SB202190 for 1 h, followed by incubation with 40 µM cyproheptadine for another 24 h in the presence of SB202190. Total cellular proteins were extracted, and protein concentration was determined for the extracts using the Bio-Rad Protein Assay reagent (Bio-Rad) with bovine serum albumin as a standard. Each lysate (10 µg) was resolved on denaturing polyacrylamide gels and transferred electrophoretically to PVDF transfer membranes. After blocking with 3% blocker (Bio-Rad) in Tris-buffered saline with Tween 20 (TBST), the membranes were incubated at room temperature for 2 h with primary antibodies—1:5000 diluted antibody against GAPDH; 1:1000 diluted antibody against p21, p27, p38 MAPK, or phospho-p38 MAPK (Thr180/Tyr182) (Cell Signaling, Danvers, MA); or 1:1000 diluted antibody against p16^INK4A^ or HBP1 (Millipore, Temecula, CA). Immunoreactive proteins were detected by incubation with horseradish peroxidase–conjugated secondary antibodies for 1 h at room temperature. After washing with TBST, the reactive bands were developed with an enhanced chemiluminescent HRP substrate detection kit (Millipore, Billerica, MA) and identified using the BioSpectrum 800 imaging system (UVP).
